# Axillary Hyperpigmentation Treatment: A Systematic Review of the Literature

**DOI:** 10.1111/jocd.70418

**Published:** 2025-08-22

**Authors:** Seyed Mohammad Vahabi, Sajjad Sajjadi, Yasamin Kalantari, Elnaz Pourgholi, Sama Heidari, Ifa Etesami

**Affiliations:** ^1^ Department of Dermatology, Razi Hospital, School of Medicine Tehran University of Medical Sciences Tehran Iran; ^2^ Department of Dermatology, Venerology and Allergology Charité Universitätsmedizin Berlin Berlin Germany

**Keywords:** axillary hyperpigmentation, PIH, post‐inflammatory hyperpigmentation, Q‐switched Nd:YAG

## Abstract

**Introduction:**

Post‐inflammatory hyperpigmentation is a common dermatologic complaint that affects different parts of the body. Axillary hyperpigmentation (AH) is a common dermatologic complaint, with no standard treatment. We aim to address the efficacy and safety of the studied treatment modalities for AH.

**Methods:**

A systematic search was done using “axillary” and “hyperpigmentation”‐related keywords/MeSH terms through PubMed/Medline, Scopus, Web of Science, and Embase.

**Results:**

Ten studies on AH were categorized into topical treatments, light/laser‐based therapies, and comparative approaches. Topical agents like niacinamide, desonide, sweet orange extract, *Perilla frutescens* leaf extract, *Cyperus rotundus* oil, glycolic acid, and novel serums demonstrated varying degrees of pigmentation reduction, with desonide and *C. rotundus* oil showing significant effectiveness and minimal side effects. Q‐switched Nd:YAG laser was effective across three studies. Both Q‐switched Nd:YAG laser and intense pulsed light (IPL) modalities showed significant improvement with minimal adverse events; though pain was more reported with the laser. A comparative study found IPL superior to alpha‐hydroxy acid in skin lightening and texture improvement. One study on Curcuma aeruginosa was excluded due to its primary focus on hair growth reduction rather than pigmentation.

**Conclusion:**

The most frequently used laser in treating AH was QS Nd‐YAG without having any severe adverse effect. Various topical treatments including Niacinamide, desonide, HQ, AHA, and a few herbal extracts were shown to be effective for AH.

## 1 Introduction

Post‐inflammatory hyperpigmentation (PIH) is a common dermatologic complaint that affects different parts of the body. An international survey showed it affects almost 15% of the population; it is more prevalent in women [1, 2]. It has prominent effects on patients' quality of life, social, and psychological status [1, 3]. Different factors such as genetics, specific drugs, inflammation, and trauma such as sun exposure or acne play a role in the hyperpigmentation process. It usually appears as hyperpigmented macules or plaques, which can affect the dermis, epidermis, or both [2, 4]. While PIH is a common cause of axillary pigmentation, it is important to consider and rule out other potential etiologies such as erythrasma, freckling, and genetically associated pigmentation disorders like neurofibromatosis type 1 and acanthosis nigricans. These conditions should receive proper treatment instead of lightening agents [5, 6].

PIH pathogenesis is not fully explained yet, but there are several factors that affect it. Higher melanogenesis or melanin deposition in response to an inflammatory process or an external injury such as UV or shaving results in PIH. Damage in the basal layer of the epidermis, besides inflammatory markers like interleukin‐1, tumor necrosis factor (TNF), and epidermal growth factor (EGF) increases transport synthesis of melanin to the keratinocytes [7].

There is no standard treatment for PIH conditions. Studies suggest that prevention by sunscreen and other methods is useful since the duration of treatment can vary up to 8 years [8]. To date, there are several treatments for PIH, including topical steroids, niacinamide, retinoids, and laser or energy‐based device interventions [9, 10]. These methods can also have adverse effects such as irritation or erythema in the affected area.

Axillary has a different tissue in comparison to other parts of the body. It has more sebaceous and sweat glands, more hair follicles, and a higher water loss value through the epidermis. This structure makes a weaker skin barrier in that part [11]. Axillary hyperpigmentation (AH) is a dermatologic complaint, especially in women with darker skin. Hyperpigmentation in the axillary is more common than in other parts of the skin because of irritation and inflammation usually caused by body folds, friction, hair plucking, shaving, deodorants, and using whitening agents [11, 12, 13]. Although there are different treatment options for skin pigmentation, there is no standard treatment for AH.

In this study, we aim to address the efficacy and safety of the studied treatment modalities for AH. This review study provides readers an overview of current treatment options for AH and helps them to make better treatment and research plans on this topic.

## 2 Materials and Methods

The aim of this systematic study is to investigate the efficacy and safety of the studied treatment modalities for AH. It follows the 2020 guidelines of the Preferred Reporting Items for Systematic Reviews and Meta‐analyses (PRISMA) [14].

### 2.1 Search Strategy

A systematic search was done using “axillary” and “hyperpigmentation”‐related keywords/MeSH terms through PubMed/Medline, Scopus, Web of Science, and Embase until June 1, 2025 (Data S1).

### 2.2 Eligibility Criteria and Study Selection

**FIGURE 1:**
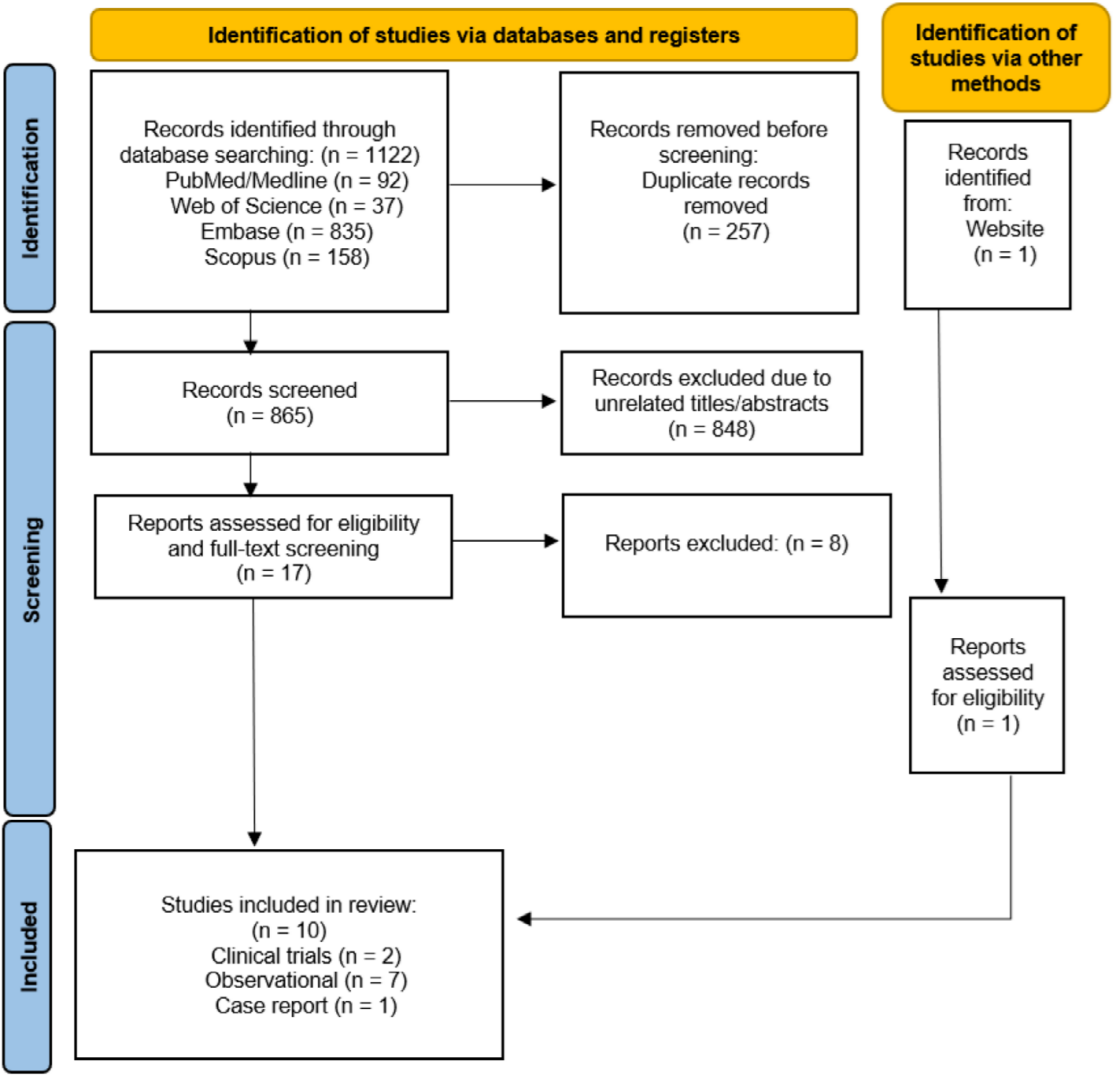
Preferred Reporting Items for Systematic Reviews and Meta‐Analyses (PRISMA) flow chart of the number of studies identified and selected into the systematic review and meta‐analysis.

The inclusion criteria were studies in which they investigated the efficacy of a treatment for AH in healthy adults. The exclusion criteria were as follows: hyperpigmentation in other areas, presence of a systemic disease, or any skin conditions that are a sign of other diseases like Acanthosis nigricans, immunocompromised patients, a history of skin cancer or keloid, and skin infection (Figure 1).

For the quality of included papers, we used the National Heart, Lung, and Blood Institute (NHLBI) quality assessment tools, and the quality of the included studies was assessed by two reviewers. This tool assesses the quality of papers in terms of methodology, report of the findings, and possible distortions based on a questionnaire checklist (Data S2 and S3).

### 2.3 Data Extraction

Four independent reviewers screened the articles to exclude irrelevant ones. In case of disagreement, a fifth reviewer made the final decision. Extracted data included study characteristics, patient age, sex, Fitzpatrick score, treatment modality, outcome measurement, outcomes, and possible adverse effects.

## 3 Results

Ten articles were enrolled (Table 1). The retrieved articles are categorized into three groups: “topical treatments,” “light‐ and laser‐based modalities,” and “comparing topical and light‐ and laser‐based modalities.”

**TABLE 1:** Characteristics of the included studies.

Study design	Population age, sex Fitzpatrick score	Treatment	Outcome measure	Outcome	Adverse effect
**Topical treatments**					
Castanedo‐Cazares et al. [15] (2013), RCT	24 females, 19–24 (Mean 21), III (21%), IV (46%), V (33%)	Niacinamide 4%, desonide 0.05%, or placebo daily every night for 9 weeks.	Tristimulus colorimeter (Chromameter CR‐300, Minolta, Osaka, Japan), histologic evaluation.	Both modalities were more effective in comparison with placebo (*p* = 0.03); However, desonide showed a better depigmenting effect than niacinamide (*p* = 0.002). A good to excellent response was observed in 30% of desonide, 24% of cases for niacinamide, and 6% for placebo.	None.
Colpas et al. [16] (2019), Observational	33 females, 18–58, NA	Patients applied the serum in one axilla and the cream to the other twice a day for 90 days. Serum containing CG‐TGP2, nicotinamide, 4‐butyl‐resorcinol, N‐undecylenoyl phenylalanine and dihydroavenanthramide compared with a cream containing nicotinamide, arbutin, bisabolol and retinaldehyde.	Dermatologic exam, photographic analysis, skin colorimetry.	Based on L colorimetric analysis, the area treated with serum showed a 62.5% improvement in skin whitening, compared to a 33.14% improvement in the area treated with cream. Overall, there was no significant difference between two products.	NA.
Preedalikit et al. [17] (2020), Observational	30 females, 20–55, III–IV	The PFLE was formulated as an underarm serum (PL serum) and applied twice daily for 4 weeks.	The efficacy was evaluated on B16F10 cell line, and healthy volunteers. Changes in skin melanin and erythema index.	A gradual decrease in melanin index from 37.94 ± 0.66 to 35.90 ± 0.64 (5.38%), and a significant decrease in erythema index from 11.32 ± 0.79 to 10.21 ± 0.11 (9.80%) were observed during a 4‐week period.	None.
Mohammed [18] (2022), RCT	153 females, 19–32, III (16%), IV (52%), V (32%)	First group received 0.25 mL *Cyperus rotundus* oil twice daily. Participants in Groups 2 (HQ) and 3 (cold cream) implemented the same amount and procedure with HQ 4% and placebo.	A tri‐stimulus colorimeter. Physician Global Assessment, self‐assessment questionnaire.	Both modalities were effective (*p* < 0.05) with similar efficacy.	82% of the HQ group reported erythema and irritation, and 48% reported itching.
Murlistyarini et al. [19] (2022), Case report	2 females, 23–24, III	During 8 weeks patients applied 8% GA cream every night and two sessions of peeling with 15% GA were performed for each.	Von Luschan's chromatic scale assessed by 3 evaluators.	The hyperpigmentation scores in first patient decreased from 23 to 14 and 20 to 10 in second patient. Both patients reported satisfactory improvements.	None.
Oei et al. [20] (2024), Observational	32 females, 18–25, NA	Patients applied one fingertip of sweet orange peels ( *Citrus sinensis* L.) 0.1% extract cream twice daily for 8 weeks and evaluation conducted every 2 weeks.	Melanin index, Physician global assessment (PGA).	The melanin index was initially 349.04 ± 109.84 AU which decreased to 253.06 ± 96.36 AU after 8 weeks of treatment. (*p* < 0.001). According to the Physician Global Assessment 68.8% of patients showed mild improvement (26%–50%) and 25% experienced good improvement (51%–75%).	None.
**Light and laser‐based modalities**					
Ghannam et al. [21] (2017), Observational	17 females, 19–48 (Mean 34.27 ± 9.24), IV–V	QS treatment was performed every 2 weeks. The number of treatments varied between 3 and 12 sessions.	Standardized grading scale.	Good improvement score of 4 ± 0.44 (51% to 75% change).	Hypopigmentation in one patient was observed.
Robredo [12] (2020), Observational	9 females, 20–52, IV–V	Four sessions of nanosecond 1064 nm Q‐switched Nd:YAG treatment every 2 weeks.	Pigmentary changes of PIH lesion compared to non‐affected regions and GIAS satisfaction assessment, pain visual analogue scale (VAS) scoring.	All axillae responded to the laser treatment, showing an 85%–100% reduction in pigmentation across the lesion area. Global Aesthetic Improvement Scale (GAIS) satisfaction ratings indicated 22%–33% of patients achieved excellent improvement, while 55% experienced significant improvement.	None.
Amornpetkul et al. [22] (2021), Observational	22 females, 20–60, III: 2 (9.1%), IV: 20 (90.9%)	Each individual received five split‐side treatments every 2 weeks.	Melanin index (MI), color chart level using the Pantone SkinToneTM Guide, im‐ provement grading scale (IGS), patient satisfaction scores.	Both treatments significantly improved after three sessions. There was no significant difference in MI, color chart level, IGS, and patient satisfaction scores between two treatments.	Regarding IPL, Hyperpigmentation in 1 (4.45%) after, erythema in 1 (4.45%). No side effects were observed in the QS‐treated group.
**Comparing topical and light and laser‐based modalities**					
Jarritrum et al. [23] (2014), Observational	20 females, 24–30 (Mean 29), III:3/20, IV: 9/20, and VI: 8/20	One underarm side was treated with 40% AHA. The other underarm side was treated with IPL wavelength of 530 nm. Patients received biweekly treatment for five times.	Skin color level of underarms, the satisfaction of volunteer.	AHA treatment enhanced skin color 65% and 35% for one level and two levels, respectively. Meanwhile, IPL treatment improved skin color 30% for one level and 70% for two levels. The improvement of skin smoothness was 5% and 45% for AHA and IPL, respectively. The evaluation of satisfaction after treatment showed that the participants in this project are pleased with IPL 15% more than AHA.	None.

Abbreviations: AHA, Alpha Hydroxy Acid; GA, Glycolic acid; HQ, hydroquinone; IPL, Intense Pulsed Light; PFLE, *Perilla frutescens* L. leaves extract; QS, Q‐switched Nd:YAG laser.

### 3.1 Topical Treatments

Six studies fell under the category of topical treatments. One study compared the effectiveness of 4% niacinamide with 0.05% desonide, while another assessed the efficacy of *Cyperus rotundus* oil in combination with hydroquinone (HQ). A third article investigated the effectiveness of *Perilla frutescens* L. leaf extract (PFLE) serum. Another study compared two novel topical formulations. One study evaluated the effects of sweet orange extract, and the final study examined the impact of glycolic acid.

In a study by Castanedo‐Cazares et al. [15] on 24 female patients (women from tropical areas including Latin America with Fitzpatrick III to V), patients randomly received niacinamide 4%, desonide 0.05%, or placebo every night for 9 weeks to treat AH. Both niacinamide and desonide demonstrated greater effectiveness compared to placebo (*p* = 0.03); however, in terms of depigmenting effect, desonide had a better effect (*p* = 0.002). A good to excellent result was observed in 30% of desonide, 24% of niacinamide, and 6% of placebo. No adverse effect was noted.

In an RCT by Mohammed et al. [18], they compared the effectiveness of *C. rotundus* essential oil (CREO) HQ 4% with placebo (cold cream). 153 participants (Fitzpatrick III to V) after 12 weeks of treatment were assessed based on colorimetric values measured by a colorimeter (Chromameter CR‐300; Minolta, Osaka, Japan). Both CREO (12.8 ± 6.1 to 7.4 ± 3.4 [*p* < 0.001]) and HQ (10.9 ± 4.7 to 7.8 ± 1.5 [*p* < 0.001]) showed better depigmenting effects than placebo (12.6 ± 4.3 to 12.8 ± 4.3 [*p* = 0.9]). No significant difference in terms of depigmentation efficacy was observed (*p* > 0.05). In terms of anti‐inflammatory and hair growth reduction effects, CREO was significantly more effective (*p* < 0.05). Regarding the adverse events, 82% of the HQ group noted erythema and irritation, and 48% noted pruritus. In contrast, none of the patients treated with CREO complained of erythema or itching. Notably, one patient (2%) reported an unpleasant smell associated with CREO.

In a study carried out by Oei et al. [20] in 2024, the extract of sweet orange was formulated into a cream. Sweet orange contains polyphenols, vitamin A, vitamin E, and ascorbic acid, and its peel exhibits an anti‐tyrosinase effect. Tyrosinase plays a main role in melanogenesis. The cream was applied twice daily to both axillae for 8 weeks and assessed every 2 weeks. At the end, the melanin index decreased from 349.04 ± 109.84 arbitrary unit (AU) to 253.06 ± 96.36 AU (*p* < 0.001). Hyperpigmentation evaluated by global physician assessment revealed 68.8% of patients showed mild improvement (26%–50%), and 25% experienced good improvement (51%–75%).

Colpas et al. [16] compared a serum containing Oligopeptide‐34 (CG‐TGP2), nicotinamide, 4‐butyl‐resorcinol, N‐undecylenoyl phenylalanine, and dihydroavenanthramide and a cream containing nicotinamide, arbutin, bisabolol, and retinaldehyde. There was no significant difference between the two; but based on L colorimetric value, the serum showed a better improvement (62.5%) (*p* < 0.001) compared to the cream (33.14%) (*p* < 0.001).

Preedalikit et al. [17] evaluated the effect of *Perilla frutescens* L. leaves extract (PFLE) serum on axillary brightening and melanogenesis in 30 Thai participants (Fitzpatrick III to V) who applied the serum twice daily for 4 weeks. A significant reduction in melanin index (37.94 ± 0.66 to 35.90 ± 0.64 (5.38%) [*p* < 0.05]) was observed; the agent improved AH without skin irritation.

Murlistyarini et al. [19] performed a case study involving two female patients with a Fitzpatrick score of III. Each patient applied 8% glycolic acid cream every night and underwent two sessions of 15% glycolic acid peeling at Weeks 2 and 6. By Week 8, the hyperpigmentation score decreased from 23 to 14 in the first patient and from 20 to 10 in the second.

### 3.2 Light and Laser‐Based Devices

Three studies were included in this category; all of which evaluated the effectiveness of the Q‐switched Nd:YAG laser, and one of these studies compared its efficacy with intense pulsed light (IPL).

In a study carried out by Ghannam et al. [21], 17 female patients (Fitzpatrick IV and V) received Q‐switched Nd:YAG laser (Spectra VRM, Lutronic, Ilsan, Gyeonggi, South Korea) every 2 weeks. The treatment protocol included a pulse repetition rate of 10 Hz, a 5 ns pulse width, an 8 mm spot size, and a fluence of 1.4 J/cm^2^, with two passes over the entire area with 20%–30% overlap. Subsequently, the laser was set on micro‐pulsed Spectra mode with a 7 mm spot size, 3 laser passes, 300 ns pulse width, and 20%–40% overlap. The results showed a good improvement in scores (4 ± 0.44; range: 4–5). In order to reach an excellent patient‐evaluated improvement, a minimum number of 3 treatment sessions was required; though increasing the number of sessions did not necessarily improve the results. Regarding adverse effects, hypopigmentation was observed in one patient.

The next study performed by Robredo et al. [12], 9 Filipino females with a Fitzpatrick score of IV to V underwent four sessions of nanosecond 1064 nm Q‐switched Nd:YAG 1064 nm (Alma Q, Alma Lasers GmbH, Nurnberg, Germany) treatment every 2 weeks. The treatment protocol consisted of four passes, a 7 mm spot size, and a fluence of 2.6 mJ/cm^2^ until total energy reached 800 kJ. All patients demonstrated good results and pigmentation reduction in 85%–100% of lesions. The Global Aesthetic Improvement Scale (GIAS) satisfaction score revealed 22%–33% excellent improvement and 55% much improved scores. No adverse effects were observed.

The last study [22] compared the efficacy of Q‐switched Nd:YAG laser (frequency of 10 Hz, fluence of 2.0–2.5 J/cm^2^, 3–5 passes, and spot size of 6 mm) with IPL (550–1200 nm, spot size 30 mm, single‐shot, and 5–8 J/cm^2^ fluence, with 1 pass treatment). Amornpetkul et al. conducted an RCT with 22 patients (Fitzpatrick III to IV), and all participants received five split‐sided therapies every 2 weeks. Both treatments demonstrated significant improvement from baseline. No significant difference was observed in the improvement grading scale (IGS) (*p* = 0.879) (2.35 ± 1.01 (25% improvement) for the IPL and 2.4 ± 1.02 (25% improvement) for the Q‐switched Nd:YAG laser). The skin color level did not differ significantly between modalities (*p* = 0.673) (8.41 ± 2.17 at baseline to 7.47 ± 1.58 for IPL and 8.27 ± 1.98 to 7.58 ± 1.57 for the Q‐switched Nd:YAG laser). There was no significant difference in the reduction in the MI for *p* = 0.811 (from 533.64 ± 28.78 at baseline to 528.37 ± 31.97 for IPL and from 533.63 ± 31.55 to 529.06 ± 29.13 for the Q‐switched Nd:YAG laser). VAS for the IPL (8.31 ± 1.7) and the QS (8.22 ± 1.7) did not differ significantly (*p* = 0.707). The QS group complained of more pain than the IPL group. In the IPL‐treated group, erythema 1 day after the therapy in one patient (4.45%) and hyperpigmentation in another patient (4.45%) 2 weeks after the second IPL were noted.

### 3.3 Comparing Topical and Light and Laser‐Based Modalities

Jarritrum et al. [23] evaluated the effectiveness of Alpha Hydroxy Acid (AHA) 40% and IPL (wavelength of 530 nm) for the treatment of AH on 20 patients receiving 5 treatments with 2‐week intervals. One underarm side was treated with AHA, and the other was treated with IPL. Regarding AHA, in 65% of patients, the skin color was enhanced by one level, and in 35% of patients, it was improved by two levels. IPL enhanced skin color by one level in 30% and by two levels in 70% of patients. Skin smoothness was enhanced by 5% and 45% for AHA and IPL, respectively.

One‐month post‐therapy, AHA revealed one level of skin color recovery in 30% and a 30% reduction in skin smoothness; while IPL showed no change.

Regarding side effects, skin redness, burning surface, and itching were most commonly observed in the group treated with AHA (100%) after the first treatment. Also, dry and flaking skin was noted in the group treated with AHA. Regarding IPL, skin redness, dry skin, and itch were reported.

It is worth mentioning that we found one clinical trial on 60 females that primarily evaluated the effect of Curcuma aeruginosa Roxb. essential oil for slowing the growth of hair and found that this treatment also lightens the axillary region. Patients received the agent for 10 weeks; after that, they received a placebo for 2 weeks [24]. Since we exclusively included the studies that primarily focused on AH treatments, this study was excluded from our manuscript.

## 4 Discussion

In this study, we found that both topical in addition to light and laser‐based therapies are effective. The studied topical agents for AH were niacinamide, desonide, CREO, AHA, HQ, *Citrus Sinensis* L., and PFLE. Light and laser therapies applied for AH were QS‐Nd YAG and IPL. Many of these treatments are being used off‐label for AH.

AH is a very common aesthetic complaint, especially in dark‐skinned women. Skin barriers are weaker in the axillary area because of higher water loss through the epidermis and the presence of more sebaceous and sweat glands. Also, using chemical deodorants, shaving, and friction cause irritation and skin tissue damage, which result in PIH [11, 22, 25]. In a study evaluating the histopathology of hyperpigmented axillae in Filipino patients, they hypothesized that darkening of the axillary is mild PIH. It is characterized by skin irritation or stimulation (mostly by hair plucking), which results in a melanin increase in the epidermis [13].

However, there exist no gold standard treatments for it. According to literature, it has been proposed that AH is a type of PIH. There exist various treatment strategies for PIH, including topical therapy, including hydroquinone, which is commonly combined with topical retinoid and steroid, as in Kligman formula, chemical peels such as Glycolic and salicylic acid, and laser therapies including Q‐switched ruby, picosecond, and Q‐switched Nd:YAG lasers [26]. Applying lasers in dermatologic diseases has a wide spectrum. Among these, we can mention hair removal, scar treatment, rejuvenation, post‐acne erythema, tattoo removal, and skin pigmentation conditions [27, 28, 29, 30].

Pablo et al. compared the effectiveness and safety of niacinamide, desonide, and placebo in treating AH in an RCT with 24 females. Niacinamide has anti‐inflammatory effects that cause depigmentation by prohibiting the melanosomes from going from the melanocyte to the keratinocyte [31]. Desonide is a synthetic, low‐potency corticosteroid and is generally considered safe. Topical corticosteroids can also reduce the PIH and cause depigmentation. The results of that study showed that both agents were effective; however, desonide had a superior blanching impact. They also proposed that desonide might decrease the inflammation involving the basal membrane disruption or stop the pathways which postpone the recovery. No change in the melanocyte count was noted, and they concluded that melanogenesis relies on enhanced melanin production and not on the cell number alterations [32].

In another study with 153 participants, the efficacy of CREO, HQ, and placebo was compared. The oil of the grass *Cyperus rotundus* (purple nutsedge) has anti‐inflammatory in addition to antipigmenting effects (as effective as HQ) and it inhibits tyrosinase activity. The authors of that study concluded that CREO had significantly better anti‐inflammatory and depigmenting effects and a decrease in hair growth compared to HQ. Cyperus rotundus has anti‐inflammatory and antioxidant effects because of its polyphenol, tannin, and flavonoid components [33, 34]. It also showed inhibitory effects on melanogenesis in B16F10 melanoma cells [35].

Another study examined the efficacy of PFLE serum ( *Perilla frutescens* L. leaves extract) on α‐melanocyte‐stimulation hormone (α‐MSH), which is a chemical involved in melanogenesis. This herbal medicine product contains a variety of potent antioxidants in leaves, having various biological effects. The results showed that this serum is effective in axillary whitening and causes a significant reduction in melanin and the erythema index in comparison to baseline and improves the skin tone [17].

Two other reports showed the efficacy of glycolic acid and sweet orange peel. Glycolic acid with modified pH showed to have anti‐inflammatory and epidermal renewal and collagen production stimulative effects [36]. Sweet orange peel has flavonoid components, which affect melanin production through their tyrosinase enzyme inhibitory nature. These components can inhibit melanogenesis [37, 38]. Also, hesperidin, another component in citrus peels, showed inhibition of melanin synthesis in melanoma B16 cells [39].

Two observational studies exclusively investigated the use of Q‐switched Nd:YAG on AH, and another study compared Q‐switched Nd:YAG with IPL. The first observational study on nine patients [12] evaluating the use of nanosecond 1064 nm Q‐switched Nd:YAG showed that 33.3% of individuals had excellent results, and 55.5% manifested significant enhancement in AH. In this regard, Ghannam et al.'s observational study on 17 females showed that low‐fluence Q‐switched 1064‐nm Nd:YAG laser is a good treatment option for AH [21]. They proposed that it leads to good‐to‐excellent enhancement after a minimum of 3 sessions. Additionally, it was concluded that the outcome was not related to increased session numbers. It has been shown that QS laser can decrease melanin granules and melanosomes and is effective for treating various pigmentation diseases, namely melasma, as revealed by histologic studies [40].

Regarding comparison of Q‐switched Nd:YAG and IPL for AH, an study on 22 participants was conducted by Amornpektul et al. [22]. The result of that study suggested that both modalities are useful with no significant difference in the result. IPL showed a lower level of pain during the procedure compare to Q‐switched Nd:YAG. A serious adverse effect of IPL, especially in patients with darker skin, is hyperpigmentation; however, it is rare [22]. Previous studies showed efficacy and safety of IPL in treatment of hyperpigmentations in other parts of the body [41, 42, 43]. Due to being cost–benefit and commonly used in dermatology, IPL seems to be a very good choice for AH.

One study [23] evaluated the efficacy of IPL and AHA on 20 subjects. AHA is a chemical peeling agent belonging to a group of organic acids. Furthermore, it has been suggested that AHA can inhibit the tyrosinase enzyme. The results of that study showed that both of these modalities are effective in lightening axillae and rejuvenating underarm skin and enhancing smoothness. However, IPL was more effective than AHA regarding whiteness (85%) and smoothness (30%), respectively. Moreover, adverse effects of AHA were more serious than those of IPL due to the high concentration (40%) of AHA and low pH of 2.

Although our study is a comprehensive review of available treatments for AH, it also has limitations. The available studies on this topic are limited, mostly with small sample sizes and short follow‐up periods. There is also a lot of variation in treatment methods, which made us unable to do a meta‐analysis. These limitations are due to the lack of standard treatment for AH and the few published studies on this topic.

In conclusion, all topical, light, and laser‐based devices are useful treatments for AH. The most frequently used laser in treating AH was QS Nd‐YAG without having any severe adverse effects. Various topical treatments, including niacinamide, desonide, HQ, AHA, and a few herbal extracts, were shown to be effective for AH. However, based on this review, very few studies are available in the literature, and larger randomized studies in this field are essentially needed. This study shed light on current available treatments, as well as giving ideas to readers to conduct new studies in the future to improve treatment outcomes and potential innovations.

## Author Contributions

Conceptualization: I.E., Y.K., S.M.V.; data curation: S.S., E.P., S.H.; methodology: S.S., E.P.; supervision: I.E., S.M.V.; validation: S.H., Y.K.; writing – original draft preparation: S.M.V., S.S., Y.K., E.P.; writing – review and editing: S.M.V., I.E., S.H. All authors have read and agreed to the published version of the manuscript.

## Conflicts of Interest

The authors declare no conflicts of interest.

## Supporting information


**Data S1:** Search query.

**Data S2:** Quality assessment E.P.

**Data S3:** Quality assessment S.S.

## Data Availability

The authors have nothing to report.
