# Supplementary material for: Axillary Hyperpigmentation Treatment: A Systematic Review of the Literature
Source: J Cosmet Dermatol. 2025 Aug 22;24(8):e70418. doi: 10.1111/jocd.70418 (PMC12371607; doi:10.1111/jocd.70418)
Supplement: Supplementary file 1 — Data S1: Search query. [file JOCD-24-e70418-s002.docx]

**6/1/2025**

PubMed/Medline: **92**

((Axillary) AND ((((((("Hyperpigmentation"[Mesh]) OR (Hypermelanosis)) OR (hypermelanosis)) OR (Post-Inflammatory Hyperpigmentation)) OR (Hyperpigmentation, Post-Inflammatory)) OR (Hyperpigmentations, Post-Inflammatory)) OR (Post Inflammatory Hyperpigmentation))) AND (((management) OR (treatment)) OR (treatments))

Scopus: **158**

((TITLE-ABS-KEY("Axillary")) AND ((((TITLE-ABS-KEY("Hyperpigmentation")) OR (TITLE-ABS-KEY("Hypermelanosis"))) OR (TITLE-ABS-KEY("hypermelanosis"))) OR (TITLE-ABS-KEY("Post-Inflammatory Hyperpigmentation"))) OR (TITLE-ABS-KEY("Hyperpigmentation, Post-Inflammatory")) OR (TITLE-ABS-KEY("Hyperpigmentations, Post-Inflammatory")) OR (TITLE-ABS-KEY("Post Inflammatory Hyperpigmentation"))) AND ((TITLE-ABS-KEY("management")) OR (TITLE-ABS-KEY("treatment")) OR (TITLE-ABS-KEY("treatments")))

Embase: **835**

(('Axillary':ab,ti) AND ((((('Hyperpigmentation':ab,ti) OR ('Hypermelanosis':ab,ti)) OR ('hypermelanosis':ab,ti)) OR ('Post-Inflammatory Hyperpigmentation':ab,ti)) OR ('Hyperpigmentation, Post-Inflammatory':ab,ti)) OR ('Hyperpigmentations, Post-Inflammatory':ab,ti)) OR ('Post Inflammatory Hyperpigmentation':ab,ti) AND ((('management':ab,ti) OR ('treatment':ab,ti)) OR ('treatments':ab,ti))

WOS: **37**

TS=(Axillary AND ("Hyperpigmentation" OR "Hypermelanosis" OR "Post-Inflammatory Hyperpigmentation" OR "Hyperpigmentation, Post-Inflammatory" OR "Hyperpigmentations, Post-Inflammatory" OR "Post Inflammatory Hyperpigmentation") AND (management OR treatment OR treatments))

All: **1122**

Duplicate: 257

Remained: 865
