# Supplementary material for: Axillary Hyperpigmentation Treatment: A Systematic Review of the Literature
Source: J Cosmet Dermatol. 2025 Aug 22;24(8):e70418. doi: 10.1111/jocd.70418 (PMC12371607; doi:10.1111/jocd.70418)
Supplement: Supplementary file 3 — Data S3: Quality assessment S.S. [file JOCD-24-e70418-s003.docx]

1. Pablo et al. 2013: Controlled Intervention Study, Good

| **Criteria** | **Yes** | **No** | **Other (CD, NR, NA)*** |
| --- | --- | --- | --- |
| 1. Was the study described as randomized, a randomized trial, a randomized clinical trial, or an RCT? | * |  |  |
| 2. Was the method of randomization adequate (i.e., use of randomly generated assignment)? | * |  |  |
| 3. Was the treatment allocation concealed (so that assignments could not be predicted)? | * |  |  |
| 4. Were study participants and providers blinded to treatment group assignment? | * |  |  |
| 5. Were the people assessing the outcomes blinded to the participants' group assignments? | * |  |  |
| 6. Were the groups similar at baseline on important characteristics that could affect outcomes (e.g., demographics, risk factors, co-morbid conditions)? | * |  |  |
| 7. Was the overall drop-out rate from the study at endpoint 20% or lower of the number allocated to treatment? | * |  |  |
| 8. Was the differential drop-out rate (between treatment groups) at endpoint 15 percentage points or lower? | * |  |  |
| 9. Was there high adherence to the intervention protocols for each treatment group? | * |  |  |
| 10. Were other interventions avoided or similar in the groups (e.g., similar background treatments)? | * |  |  |
| 11. Were outcomes assessed using valid and reliable measures, implemented consistently across all study participants? | * |  |  |
| 12. Did the authors report that the sample size was sufficiently large to be able to detect a difference in the main outcome between groups with at least 80% power? |  |  | NR |
| 13. Were outcomes reported or subgroups analyzed prespecified (i.e., identified before analyses were conducted)? | * |  |  |
| 14. Were all randomized participants analyzed in the group to which they were originally assigned, i.e., did they use an intention-to-treat analysis? | * |  |  |

1. Colpas et al. 2019: Before-After (Pre-Post) Study With No Control Group: Good

| **Criteria** | **Yes** | **No** | **Other (CD, NR, NA)*** |
| --- | --- | --- | --- |
| 1. Was the study question or objective clearly stated? | * |  |  |
| 2. Were eligibility/selection criteria for the study population prespecified and clearly described? | * |  |  |
| 3. Were the participants in the study representative of those who would be eligible for the test/service/intervention in the general or clinical population of interest? | * |  |  |
| 4. Were all eligible participants that met the prespecified entry criteria enrolled? |  |  | NR |
| 5. Was the sample size sufficiently large to provide confidence in the findings? |  | * |  |
| 6. Was the test/service/intervention clearly described and delivered consistently across the study population? | * |  |  |
| 7. Were the outcome measures prespecified, clearly defined, valid, reliable, and assessed consistently across all study participants? | * |  |  |
| 8. Were the people assessing the outcomes blinded to the participants' exposures/interventions? |  | * |  |
| 9. Was the loss to follow-up after baseline 20% or less? Were those lost to follow-up accounted for in the analysis? | * |  |  |
| 10. Did the statistical methods examine changes in outcome measures from before to after the intervention? Were statistical tests done that provided p values for the pre-to-post changes? | * |  |  |
| 11. Were outcome measures of interest taken multiple times before the intervention and multiple times after the intervention (i.e., did they use an interrupted time-series design)? | * |  |  |
| 12. If the intervention was conducted at a group level (e.g., a whole hospital, a community, etc.) did the statistical analysis take into account the use of individual-level data to determine effects at the group level? |  |  | NA |

1. Preedalikit et al. 2020: Before-After (Pre-Post) Study With No Control Group: Good

| **Criteria** | **Yes** | **No** | **Other (CD, NR, NA)*** |
| --- | --- | --- | --- |
| 1. Was the study question or objective clearly stated? | * |  |  |
| 2. Were eligibility/selection criteria for the study population prespecified and clearly described? | * |  |  |
| 3. Were the participants in the study representative of those who would be eligible for the test/service/intervention in the general or clinical population of interest? | * |  |  |
| 4. Were all eligible participants that met the prespecified entry criteria enrolled? |  |  | NR |
| 5. Was the sample size sufficiently large to provide confidence in the findings? |  | * |  |
| 6. Was the test/service/intervention clearly described and delivered consistently across the study population? | * |  |  |
| 7. Were the outcome measures prespecified, clearly defined, valid, reliable, and assessed consistently across all study participants? | * |  |  |
| 8. Were the people assessing the outcomes blinded to the participants' exposures/interventions? |  | * |  |
| 9. Was the loss to follow-up after baseline 20% or less? Were those lost to follow-up accounted for in the analysis? | * |  |  |
| 10. Did the statistical methods examine changes in outcome measures from before to after the intervention? Were statistical tests done that provided p values for the pre-to-post changes? | * |  |  |
| 11. Were outcome measures of interest taken multiple times before the intervention and multiple times after the intervention (i.e., did they use an interrupted time-series design)? | * |  |  |
| 12. If the intervention was conducted at a group level (e.g., a whole hospital, a community, etc.) did the statistical analysis take into account the use of individual-level data to determine effects at the group level? |  |  | NA |

1. Robredo IGC, 2020: Before-After (Pre-Post) Study With No Control Group: Fair

| **Criteria** | **Yes** | **No** | **Other (CD, NR, NA)*** |
| --- | --- | --- | --- |
| 1. Was the study question or objective clearly stated? | * |  |  |
| 2. Were eligibility/selection criteria for the study population prespecified and clearly described? | * |  |  |
| 3. Were the participants in the study representative of those who would be eligible for the test/service/intervention in the general or clinical population of interest? | * |  |  |
| 4. Were all eligible participants that met the prespecified entry criteria enrolled? |  |  | NR |
| 5. Was the sample size sufficiently large to provide confidence in the findings? |  | * |  |
| 6. Was the test/service/intervention clearly described and delivered consistently across the study population? | * |  |  |
| 7. Were the outcome measures prespecified, clearly defined, valid, reliable, and assessed consistently across all study participants? | * |  |  |
| 8. Were the people assessing the outcomes blinded to the participants' exposures/interventions? |  | * |  |
| 9. Was the loss to follow-up after baseline 20% or less? Were those lost to follow-up accounted for in the analysis? | * |  |  |
| 10. Did the statistical methods examine changes in outcome measures from before to after the intervention? Were statistical tests done that provided p values for the pre-to-post changes? | * |  |  |
| 11. Were outcome measures of interest taken multiple times before the intervention and multiple times after the intervention (i.e., did they use an interrupted time-series design)? |  | * |  |
| 12. If the intervention was conducted at a group level (e.g., a whole hospital, a community, etc.) did the statistical analysis take into account the use of individual-level data to determine effects at the group level? |  |  | NA |

1. Amornpetkul et al. 2021: Before-After (Pre-Post) Study With No Control Group: Good

| **Criteria** | **Yes** | **No** | **Other (CD, NR, NA)*** |
| --- | --- | --- | --- |
| 1. Was the study question or objective clearly stated? | * |  |  |
| 2. Were eligibility/selection criteria for the study population prespecified and clearly described? | * |  |  |
| 3. Were the participants in the study representative of those who would be eligible for the test/service/intervention in the general or clinical population of interest? | * |  |  |
| 4. Were all eligible participants that met the prespecified entry criteria enrolled? | * |  |  |
| 5. Was the sample size sufficiently large to provide confidence in the findings? |  | * |  |
| 6. Was the test/service/intervention clearly described and delivered consistently across the study population? | * |  |  |
| 7. Were the outcome measures prespecified, clearly defined, valid, reliable, and assessed consistently across all study participants? | * |  |  |
| 8. Were the people assessing the outcomes blinded to the participants' exposures/interventions? | * |  |  |
| 9. Was the loss to follow-up after baseline 20% or less? Were those lost to follow-up accounted for in the analysis? | * |  |  |
| 10. Did the statistical methods examine changes in outcome measures from before to after the intervention? Were statistical tests done that provided p values for the pre-to-post changes? | * |  |  |
| 11. Were outcome measures of interest taken multiple times before the intervention and multiple times after the intervention (i.e., did they use an interrupted time-series design)? | * |  |  |
| 12. If the intervention was conducted at a group level (e.g., a whole hospital, a community, etc.) did the statistical analysis take into account the use of individual-level data to determine effects at the group level? |  |  | NA |

1. Mohammed et al. 2021: Controlled Intervention Study, Good

| **Criteria** | **Yes** | **No** | **Other (CD, NR, NA)*** |
| --- | --- | --- | --- |
| 1. Was the study described as randomized, a randomized trial, a randomized clinical trial, or an RCT? | * |  |  |
| 2. Was the method of randomization adequate (i.e., use of randomly generated assignment)? | * |  |  |
| 3. Was the treatment allocation concealed (so that assignments could not be predicted)? | * |  |  |
| 4. Were study participants and providers blinded to treatment group assignment? | * |  |  |
| 5. Were the people assessing the outcomes blinded to the participants' group assignments? | * |  |  |
| 6. Were the groups similar at baseline on important characteristics that could affect outcomes (e.g., demographics, risk factors, co-morbid conditions)? | * |  |  |
| 7. Was the overall drop-out rate from the study at endpoint 20% or lower of the number allocated to treatment? | * |  |  |
| 8. Was the differential drop-out rate (between treatment groups) at endpoint 15 percentage points or lower? | * |  |  |
| 9. Was there high adherence to the intervention protocols for each treatment group? | * |  |  |
| 10. Were other interventions avoided or similar in the groups (e.g., similar background treatments)? | * |  |  |
| 11. Were outcomes assessed using valid and reliable measures, implemented consistently across all study participants? | * |  |  |
| 12. Did the authors report that the sample size was sufficiently large to be able to detect a difference in the main outcome between groups with at least 80% power? | * |  |  |
| 13. Were outcomes reported or subgroups analyzed prespecified (i.e., identified before analyses were conducted)? | * |  |  |
| 14. Were all randomized participants analyzed in the group to which they were originally assigned, i.e., did they use an intention-to-treat analysis? | * |  |  |

1. Oei et al. 2024: Before-After (Pre-Post) Study With No Control Group: Good

| **Criteria** | **Yes** | **No** | **Other (CD, NR, NA)*** |
| --- | --- | --- | --- |
| 1. Was the study question or objective clearly stated? | * |  |  |
| 2. Were eligibility/selection criteria for the study population prespecified and clearly described? | * |  |  |
| 3. Were the participants in the study representative of those who would be eligible for the test/service/intervention in the general or clinical population of interest? | * |  |  |
| 4. Were all eligible participants that met the prespecified entry criteria enrolled? | * |  |  |
| 5. Was the sample size sufficiently large to provide confidence in the findings? | * |  |  |
| 6. Was the test/service/intervention clearly described and delivered consistently across the study population? | * |  |  |
| 7. Were the outcome measures prespecified, clearly defined, valid, reliable, and assessed consistently across all study participants? | * |  |  |
| 8. Were the people assessing the outcomes blinded to the participants' exposures/interventions? |  | * |  |
| 9. Was the loss to follow-up after baseline 20% or less? Were those lost to follow-up accounted for in the analysis? | * |  |  |
| 10. Did the statistical methods examine changes in outcome measures from before to after the intervention? Were statistical tests done that provided p values for the pre-to-post changes? | * |  |  |
| 11. Were outcome measures of interest taken multiple times before the intervention and multiple times after the intervention (i.e., did they use an interrupted time-series design)? | * |  |  |
| 12. If the intervention was conducted at a group level (e.g., a whole hospital, a community, etc.) did the statistical analysis take into account the use of individual-level data to determine effects at the group level? |  |  | NA |

1. Ghannam, 2017: Before-After (Pre-Post) Study With No Control Group: Good

| **Criteria** | **Yes** | **No** | **Other (CD, NR, NA)*** |
| --- | --- | --- | --- |
| 1. Was the study question or objective clearly stated? | * |  |  |
| 2. Were eligibility/selection criteria for the study population prespecified and clearly described? | * |  |  |
| 3. Were the participants in the study representative of those who would be eligible for the test/service/intervention in the general or clinical population of interest? | * |  |  |
| 4. Were all eligible participants that met the prespecified entry criteria enrolled? | * |  |  |
| 5. Was the sample size sufficiently large to provide confidence in the findings? |  | * |  |
| 6. Was the test/service/intervention clearly described and delivered consistently across the study population? | * |  |  |
| 7. Were the outcome measures prespecified, clearly defined, valid, reliable, and assessed consistently across all study participants? | * |  |  |
| 8. Were the people assessing the outcomes blinded to the participants' exposures/interventions? |  | * |  |
| 9. Was the loss to follow-up after baseline 20% or less? Were those lost to follow-up accounted for in the analysis? | * |  |  |
| 10. Did the statistical methods examine changes in outcome measures from before to after the intervention? Were statistical tests done that provided p values for the pre-to-post changes? | * |  |  |
| 11. Were outcome measures of interest taken multiple times before the intervention and multiple times after the intervention (i.e., did they use an interrupted time-series design)? | * |  |  |
| 12. If the intervention was conducted at a group level (e.g., a whole hospital, a community, etc.) did the statistical analysis take into account the use of individual-level data to determine effects at the group level? |  |  | NA |

1. Jarritrum, 2014: Before-After (Pre-Post) Study With No Control Group: Good

| **Criteria** | **Yes** | **No** | **Other (CD, NR, NA)*** |
| --- | --- | --- | --- |
| 1. Was the study question or objective clearly stated? | * |  |  |
| 2. Were eligibility/selection criteria for the study population prespecified and clearly described? | * |  |  |
| 3. Were the participants in the study representative of those who would be eligible for the test/service/intervention in the general or clinical population of interest? | * |  |  |
| 4. Were all eligible participants that met the prespecified entry criteria enrolled? | * |  |  |
| 5. Was the sample size sufficiently large to provide confidence in the findings? |  | * |  |
| 6. Was the test/service/intervention clearly described and delivered consistently across the study population? | * |  |  |
| 7. Were the outcome measures prespecified, clearly defined, valid, reliable, and assessed consistently across all study participants? | * |  |  |
| 8. Were the people assessing the outcomes blinded to the participants' exposures/interventions? |  | * |  |
| 9. Was the loss to follow-up after baseline 20% or less? Were those lost to follow-up accounted for in the analysis? | * |  |  |
| 10. Did the statistical methods examine changes in outcome measures from before to after the intervention? Were statistical tests done that provided p values for the pre-to-post changes? | * |  |  |
| 11. Were outcome measures of interest taken multiple times before the intervention and multiple times after the intervention (i.e., did they use an interrupted time-series design)? |  | * |  |
| 12. If the intervention was conducted at a group level (e.g., a whole hospital, a community, etc.) did the statistical analysis take into account the use of individual-level data to determine effects at the group level? |  |  | NA |
